# Hemolysis contributes to anemia during long-duration space flight

**DOI:** 10.1038/s41591-021-01637-7

**Published:** 2022-01-14

**Authors:** Guy Trudel, Nibras Shahin, Timothy Ramsay, Odette Laneuville, Hakim Louati

**Affiliations:** 1 Bone and Joint Research Laboratory, Ottawa Hospital Research Institute, Ottawa, Ontario Canada; 2 Division of Physical Medicine and Rehabilitation, Department of Medicine and Department of Biochemistry, Microbiology and Immunology, Faculty of Medicine, University of Ottawa, Ottawa, Ontario Canada; 3 School of Epidemiology and Public Health, University of Ottawa, Ottawa, Ontario Canada; 4 Department of Biology, Faculty of Science, University of Ottawa, Ottawa, Onatrio Canada

**Keywords:** Anaemia, Diagnostic markers

## Abstract

Anemia in astronauts has been noted since the first space missions, but the mechanisms contributing to anemia in space flight have remained unclear. Here, we show that space flight is associated with persistently increased levels of products of hemoglobin degradation, carbon monoxide in alveolar air and iron in serum, in 14 astronauts throughout their 6-month missions onboard the International Space Station. One year after landing, erythrocytic effects persisted, including increased levels of hemolysis, reticulocytosis and hemoglobin. These findings suggest that the destruction of red blood cells, termed hemolysis, is a primary effect of microgravity in space flight and support the hypothesis that the anemia associated with space flight is a hemolytic condition that should be considered in the screening and monitoring of both astronauts and space tourists.

## Abstract (web summary)

Biomarkers of red blood cell destruction were elevated in astronauts while on long-duration missions on the International Space Station, suggesting that hemolysis is a major contributor to space anemia.

## Main

As humankind plans extraterrestrial travel, understanding the health implications of living in space will be critical to planning safe journeys. Space anemia was previously documented and characterized by a 10–12% decrease in red blood cell (RBC) mass happening in the first 10 days in space^1^. Current understanding of space anemia is that the decrease in RBCs constitutes an acute adaptation to major hemodynamic events of cephalad fluid shifts, hemoconcentration and low erythropoietin (EPO) levels upon entering microgravity^1,2^. Thereafter, beyond 10 days in space, when the hemoglobin concentration returns to near-earthly values, erythrocytic regulation would proceed normally, but this has not been measured precisely^2^. Recently, astronauts were found to remain mildly hemoconcentrated throughout long-duration mission^3^, and epidemiological data showed that the severity, time to recovery and longitudinal effects of postflight anemia were proportional to the time spent in space^4^. These reports challenged the current understanding of space anemia. Longer missions to the moon and Mars, as well as space tourism and commercialization, require a better understanding of space-induced anemia. Because astronaut orthostatism, exercise tolerance and fatigue are key functions affected by anemia, RBC management will be vital for human missions landing on extraterrestrial worlds without medical supervision.

While a variety of hypothetical causes (e.g., RBC dysfunction, decreased production, sequestration or increased destruction) have been proposed for space anemia, the physiologic mechanisms are not fully established^5^, and studying these mechanisms in space is challenging. Hemolysis releases hemoglobin, and heme rings are broken down by heme oxygenases^6^. Each heme molecule produces one ferrous iron, one carbon monoxide (CO) and one biliverdin molecule. In basal conditions, approximately 85% of endogenously produced CO arises from hemoglobin^6^. The quantification of CO molecules eliminated is therefore a direct measure of hemolysis. Recently developed methods to precisely quantify endogenous CO now permit the measurement of hemolysis in space^7^. Using these methods, 20 participants showed increased hemolysis (by an average of 23%) throughout 60 days of the antiorthostatic bed-rest microgravity analogue^8^. These findings suggested that increased hemolysis may be an important primary effect of the microgravity analogue, a hypothesis never tested in space. We therefore measured hemolysis markers in breath and blood samples from astronauts preflight, four times inflight and up to 1 year after their 6-month missions to the International Space Station (ISS).

**Fig. 1:**
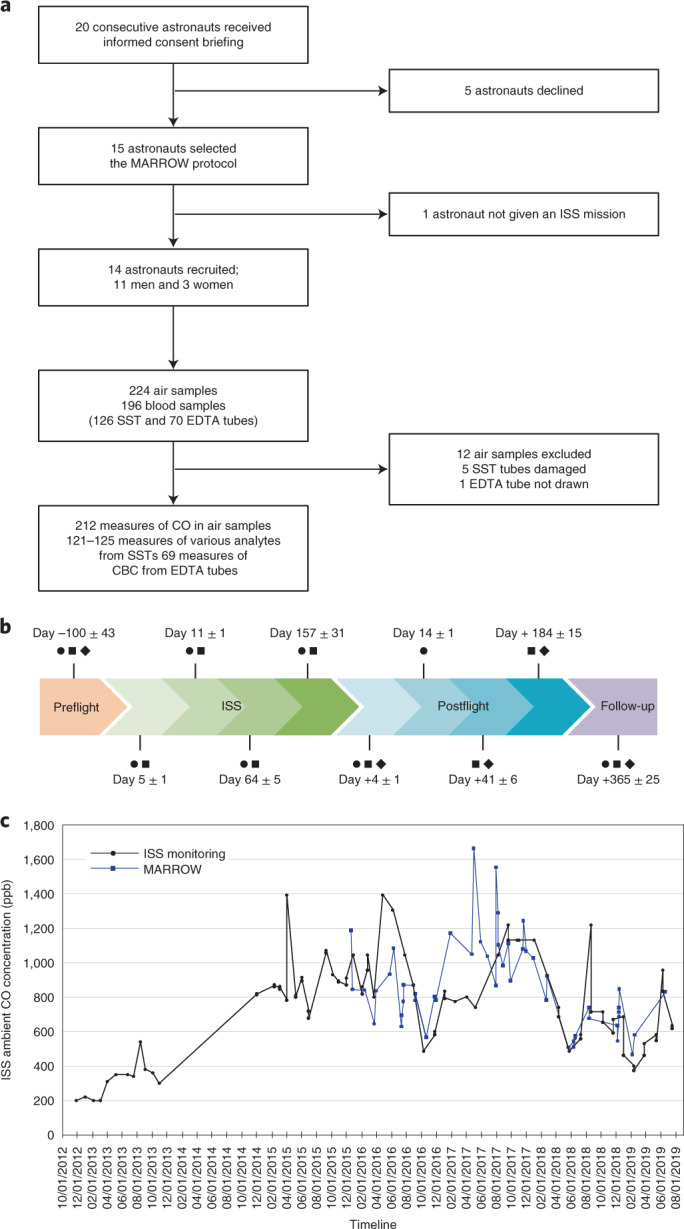
Bone Marrow Adipose Reaction: Red Or White (MARROW) study design and outcomes. **a**, Flow chart showing recruitment and samples harvested. SST, serum separator tube. **b**, Schematic showing the study design and planned sample collection. Astronauts recruited to the MARROW study performed serial measures at prespecified time points preflight, onboard the ISS and up to 1 year postflight. The average number of days (±s.d.) of sampling in relation to takeoff and landing are indicated. The interval between the last CO sampling in space and the first sampling on Earth after landing was 14 ± 5 days. Circles, CO measurements; squares, serum analytes (iron, transferrin percent saturation, ferritin, haptoglobin, bilirubin, C-reactive protein and EPO); diamonds, complete blood counts (CBCs) and reticulocytes. **c**, Time course of ambient air CO concentration onboard the ISS.

A total of 14 astronauts were recruited (11 men and 3 women; 45 ± 7 years) between 2015 and 2020 (Fig. 1a). The astronauts flew ISS missions of 167 ± 31 days duration. Each astronaut collected alveolar and ambient air samples as well as blood samples according to a prespecified schedule (Fig. 1b,c). The space samples were downmassed using automated reentry vehicles.

**Fig. 2:**
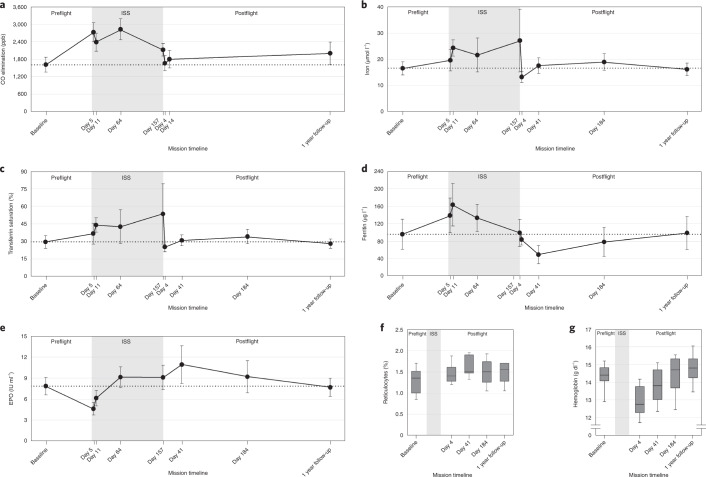
Hemolysis and erythropoietic markers of astronauts preflight, onboard the ISS and postflight. **a**, CO elimination. **b**–**e**, Serum iron concentrations (**b**), transferrin saturation (**c**), ferritin concentrations (**d**) and EPO concentrations (**e**). Data points and error bars represent mean values with ±95% CIs. Shaded areas represent data from ISS samples. **f**,**g**, Percentage of reticulocytes (**f**) and hemoglobin concentrations (**g**). The center line in the box plots represents the median, the upper limit of the box plots represents the third quartile (75th percentile), the lower limit of the box plots represents the first quartile (25th percentile), the upper whisker is the maximum value of the data that are within 1.5 times the interquartile range over the 75th percentile and the lower whisker is the minimum value of the data that are within 1.5 times the interquartile range under the 25th percentile. *N* = 14 astronauts who provided individual samples at five, eight or nine independent time points according to the outcome.

Figure 2a shows that mean baseline CO elimination was 1,662 ppb (95% confidence interval (CI), 1,433–1,891). After an average of 5 ± 1days on the ISS, CO elimination was 2,627 ppb (95% CI, 2,342–2,913); the mean increase from preflight was 56% (95% CI, 36–76; Supplementary Table 1). Increased hemolysis may have started earlier than day 5 (the first sampling time). The finding of increased CO elimination was corroborated by the observation that serum iron and iron carrier proteins transferrin and ferritin also increased (Fig. 2b–d). Large elevations in heme degradation products both in alveolar air and blood samples constitute the first direct demonstration of upregulated hemolysis in space and support the hypothesis that space-related anemia is a hemolytic condition.

The next question was whether the hemolysis stopped when the hemoglobin concentration reached near-earthly values after 10 days in space. CO elimination in space over 6 months (average of 46 measures) was increased 54% (95% CI, 39–70) compared to preflight (Fig. 2a; Supplementary Tables 1 and 2). Figure 2b–d shows that serum iron, transferrin saturation and ferritin levels in space (average of 55 measures each) were also higher compared to preflight throughout the 6-month missions (Supplementary Tables 1 and 2). Persistently increased CO elimination in space showed that hemolysis was not an acute adaptation to hemodynamic alterations upon entering microgravity. Increased hemolysis rather constituted a primary effect of exposure to space. This conclusion is strengthened by the postflight data. The astronaut first postflight measure (taken 4 days after landing, separated by only 14 days from the last inflight measure) showed sharp decreases in hemolysis markers coinciding with the return to Earth’s surface gravity (Fig. 2a–d). The pattern of hemolysis in the three phases of the study (on Earth, in space and back on Earth) confirmed that increased hemolysis was tightly linked to the space environment.

Increased hemolysis as a primary effect of exposure to space constitutes a paradigm shift in our understanding of space anemia^5^. Major pathophysiological implications include that space anemia may be dependent on the duration of exposure to space and independent of fluid shifts, EPO levels and the RBC production environment on Earth versus in space and that it may trigger a compensatory increase in RBC production and enhance nutritional needs. Persistent hemolysis during space missions suggests that the longer the exposure, the worse the anemia. A similar conclusion was reached after analyzing data from 711 missions from American and Canadian astronauts between 1968 and 2015 (ref. ^4^). Increased hemolysis was measured both during fluid shifts (inflight days 5 and 11) and long after fluid shifts were completed (inflight days 64 and 157), dissociating it from a causation effect. Increased hemolysis appeared EPO independent. In the current trial, increased hemolysis was sustained under both suppressed (inflight days 5 and 11) and corrected EPO levels (inflight days 64 and 157; Fig. 2e). In the bone marrow, colony-forming unit erythroid cells depend on EPO for survival and differentiation into erythroblasts, with only the latter synthesizing hemoglobin^9^. Reticulocytes and mature RBCs lack EPO receptors and cease responding to EPO^9^. Colony-forming unit erythroid cell suppression under low-EPO conditions should not enhance heme degradation, whereas degradation of erythroblasts, reticulocytes and mature RBCs would. Therefore, our data support increased hemolysis of EPO-independent erythroblasts and/or more differentiated RBCs. Upon entering space, all astronaut RBCs are terrestrial born. Based on normative RBC lifespan, after 120 days in space, all RBCs are space born. In this study, both RBC populations hemolyzed at higher rates while in space. At postflight day 4, the hemolysis of space-born RBCs sharply decreased. These results suggest that intrinsic characteristics of Earth-born or space-born RBCs do not influence hemolysis. Space hemolysis attenuates the initial hemoconcentration caused by fluid shifts obviating the need for compensatory RBC production. Later in the mission, a continuing 54% increased hemolysis risks causing severe anemia. That astronauts remain mildly hemoconcentrated throughout long-duration space missions^3^ implies a compensatory increased RBC production. Enhanced RBC production in space was not directly measured in this study, but robust EPO levels at inflight days 64 and 157 and increased reticulocytes at postflight day 4 supported stimulated RBC production (Fig. 2e,f and Supplementary Table 2). Hemolysis with compensatory RBC production will expand bone marrow erythropoietic activity, the higher energy requirements of which must be factored in astronaut nutritional planning. C-reactive protein levels remained unchanged throughout ISS missions, arguing against an inflammatory component to space anemia from background inflammation in space or free hemoglobin as a potent inflammatory inducer (Supplementary Tables 1–3).

The return to Earth’s surface also causes major hemodynamic changes, as fluid shift reversal increases plasma volume and dilutes RBCs^1,5^. Our astronaut cohort had an 8.8% (95% CI, 8.1–9.5) decrease in hemoglobin concentration 4 days after landing compared to preflight (Fig. 2g and Supplementary Table 2) with 5 of 13 astronauts (38.5%) reaching clinical levels of anemia^10^. Low hemoglobin concentrations postflight indicate that astronauts’ first landing measure, just like inflight measures past 10 days in space, originated from fewer RBCs^1^. These predicted proportionately lower CO elimination, iron and iron carrier protein concentrations. The implication is that the hemolysis figures we report compared to preflight data, as high as they are, are likely underestimating the actual rate of hemolysis at these time points.

The next question was how reversible these effects were up to 1 year after long-duration missions. Erythrocytic effects of space include increased hemolysis (herein reported), correspondingly elevated RBC production that is assumed and previously reported hemoglobin reset at higher concentrations^3^. Previous spaceflight data suggested persistent erythrocytic effects with higher hemoglobin concentrations (0.27 g dl^−1^) in 58 astronauts 1 year after missions averaging 145 days^4^. In the current trial, 1 year after landing, CO elimination was 30% (95% CI, 2–57) higher, reticulocytes were 16% (95% CI, 7–24) higher and hemoglobin concentration was 3.5% (95% CI, 1.5–5.4) (0.50 g dl−1) higher than preflight levels (Fig. 2a,f,g and Supplementary Tables 1 and 2). These data support increased hemolysis, RBC production and hemoglobin concentration 1 year after long-duration space exposure. These erythrocytic space effects may not be permanent, as longitudinal astronaut data over 20 years predicted lower lifelong hemoglobin concentrations (−0.001 and −0.002 g dl^−1^ for male and female astronauts, respectively) for every day in microgravity^4^. Erythrocytic effects 1 year postflight support long-term monitoring and consideration on the optimal interval between repeated space missions.

The anatomic site and mechanism of space hemolysis remain unknown. Hemolysis can be intravascular or extravascular in the spleen, liver or bone marrow. Increased heme degradation without haptoglobin depletion in this trial argues against intravascular hemolysis (Supplementary Tables 1 and 2). Erythroblast and reticulocyte destruction in the bone marrow before their egress into the circulation (sometimes called inefficient erythropoiesis) could arise from marrow adipose tissue accumulation from the lack of bone stimulation in space and dyserythropoiesis^11^. Altered size and shape of erythrocytes or the spleen in space may increase extravascular hemolysis^12^. Mature RBC lifespan may be shortened by mitochondrial stress and dysregulation^13^. Gene modifiers (e.g., BCL11A and KLF1) modulate the expression of globin genes^14^. The regulation of gene expression of these globin modifiers involves the methylation state of the DNA regions impacting their expression^15^. DNA hypomethylation in the β-globin gene cluster was linked to ineffective erythropoiesis in thalassemia^15^. Interestingly, altered methylation levels in CD4 and CD8 lymphocytes were reported after 1 year in space^2^. Space or radiation effects on DNA methylation of regulatory regions controlling the expression of globin and gene modifiers constitute potential mechanisms of space hemolysis. Elucidating the site and mechanism of space hemolysis is crucial to developing successful mitigation strategies. The specific exercise and nutritional countermeasures of modern space travel did not prevent hemolysis and postflight anemia in our cohort, and both could have health consequences. Increased CO levels from space hemolysis can have second-messenger modulatory effects on intracellular processes of the cardiac, vascular, ocular, bone, nutrition, circadian cycle, orthostatic hypotension, brain and muscle systems of astronauts^16,17^.

Space tourism will considerably expand the number of space travelers^18^. Medical screening of future astronauts and space tourists might benefit from a preflight profiling of globin gene and modifiers. Postlanding monitoring should cover conditions affected by anemia and hemolysis. Monitoring individual astronaut’s levels of hemolysis during mission may be indicated to reduce health risks^18^. Our results support adding space travel to the list of etiologies for hemolytic anemia.

Erythrokinetic response to microgravity may have critical relevance to understand erythroid physiology and pathophysiology in contexts other than space flight. Data from the mid-1990s focusing on the acute hemoconcentration entering space led to hypotheses on normal erythroid mass control, prevention from becoming excessive, hypoxic environments, polycythemia and EPO withdrawal^5^. Preferential hemolysis of younger erythrocytes after descent from altitude is currently debated^19,20^. Our findings of continuous hemolysis in space carry broad implications for populations on Earth with increased hemolysis or high prevalence of unexplained anemia, including patients receiving critical care, patients with chronic anemia, older patients, sedentary patients and bedridden patients^21–23^. Like astronauts, the environment of these individuals is characterized by a marked decrease in skeleton stimulation and decreased standing time. These individuals experience cardiovascular deconditioning, muscle atrophy and osteopenia. Potential alterations in bone marrow and spleen functions in space may be relevant to pathophysiological changes in their erythrocytic control. Our findings support investigating whether increased hemolysis contributes to anemia on Earth and could be targeted by specific physical rehabilitation strategies.

Limitations of this study include potential confounding factors associated with exposure to space, including fluid shifts, ambient CO concentration, altered circadian cycle and muscle atrophy. These factors were addressed with ambient CO sampling with each alveolar sample, chronostandardization and serial measures throughout ISS missions (Fig. 1c). Hemolysis with longer space exposure, such as 1-year ISS or Moon missions or journeys to Mars, and cumulative exposure constitute knowledge gaps. Recruitment was limited to astronauts, which threatens the generalization of these findings to space tourists. The unequal sex distribution precluded a robust examination of potential physiological differences (Supplementary Tables 3 and 4).

RBC regulation demonstrated major disruptions in space compared to on Earth. Increased hemolysis by 54% was a primary effect of exposure to space in astronauts that persisted throughout their long-duration missions and may constitute the leading mechanism of space-related anemia. Space hemolysis should be considered in the screening, monitoring and follow-up of astronauts launching on space exploration missions, as well as space tourists.

## Methods

### Design and exposure

Between 2015 and 2020, astronauts were consecutively interviewed approximatively 1 year ahead of their ISS missions. The participating astronauts collected air and blood samples 3 months before Soyuz takeoff, four times onboard the ISS and serially after landing (total of 224 air and 196 blood samples; Fig. 1a,b). Each individual astronaut could have elected to participate in a complement of different experiments, some of which might have included additional venipunctures, causing blood and iron losses. To our knowledge, astronauts in this cohort were not screened for or confirmed to not be carrying any hemoglobinopathy or one of 1,000 known β-globin variants, some of which may affect hemoglobin levels in vivo (https://globin.bx.psu.edu/hbvar/)^24,25^. In this report, preflight measures served as the comparator to test the effects of exposure to space and determine whether one or more individual astronauts unknowingly had a β-globin variant (https://globin.bx.psu.edu/hbvar/).

### Outcomes

The primary outcome was CO elimination quantified as the concentration of CO gas molecules [CO] in parts per billion eliminated through pulmonary ventilation. Increased CO elimination can arise from RBC destruction (intravascular or extravascular) in the spleen, liver or bone marrow. Clinically, whereas the first three conditions are called hemolysis, the latter is sometimes referred to as intramedullary hemolysis or ineffective erythropoiesis. In this paper, we defined, measured and discussed hemolysis as heme degradation products of the heme oxygenase enzymes arising from all sites. Secondary outcomes included other direct (iron) and indirect (bilirubin, transferrin percent saturation and ferritin) markers of heme degradation as well as haptoglobin, EPO, CBC and C-reactive protein^26,27^. CBC, reticulocytes and erythrocyte sedimentation rate (ESR) were not measured during flight, as they require analyses on fresh whole blood. In addition, measuring CBC and reticulocytes requires a hemocytometer, and measuring ESR requires gravity, both of which are unavailable on the ISS. In this trial, we did not measure the age of erythrocytes to comment on the subpopulation of RBCs present in space.

### Laboratory analyses

Various markers of increased erythrocyte destruction intravascular or extravascular are used clinically, including the presence of schistocytes on a blood smear, free hemoglobin in blood, decreased haptoglobin for its high affinity with free hemoglobin, increased bilirubin (indirect or direct), increased ferritin, increased LDH, d-dimer and hemosideruria^28^. However, for different reasons, these markers are not precise, quantitative or direct markers of hemolysis^28^. Iron and transferrin saturation may rise with many types of anemia, including hypoproliferation/hypoplasia when iron is not cleared from the plasma. Similarly, serum ferritin can increase with any cause of anemia other than iron deficiency and/or bleeding. The iron in circulating RBCs is transferred to reticuloendothelial stores, which release a proportional amount of ferritin. For these reasons, we chose endogenous CO elimination as a precise, direct and quantitative marker of hemolysis as the primary outcome measure of the study. We measured CO elimination following the methods of Shahin et al.^7^. Briefly, upon waking up, after a 20-s breath hold, astronauts exhaled through a mouthpiece connected to a one-way valve sequentially discarding 400 ml of air from airways and filling a 750-ml collection bag with alveolar air (Supplementary Video 1). The alveolar air was immediately transferred to a vacuumed 200-ml canister. Concurrently, the astronauts filled a second canister with ambient air. Canisters sampled on the ISS were stowed and boarded the next Earth-bound reentry vehicle. These methods accommodated for changes in pressure, temperature and vibration and maintained stable [CO] for at least 11 months^7^. Both air samples were extracted from the canisters and run through a gas chromatograph with a reduction gas detector. Subtracting the ambient air [CO] from the alveolar air [CO] provided the CO elimination.

### Methods for secondary outcomes

The astronauts serially drew blood in tubes with double-polymer gel (serum separator tube) (Fig. 1). Blood was centrifuged at 1,515 *g* for 10 min, and the tubes were frozen at −80 °C. For reentry, they were loaded into the −80 °C freezer aboard the Dragon capsule and processed using commercial clinical analyzers for iron, transferrin percent saturation, ferritin, haptoglobin, bilirubin, EPO and C-reactive protein (Supplementary Table 5). Blood was also drawn pre- and postflight in EDTA tubes for CBC and reticulocytes at Johnson Space Center (JSC) clinical laboratory.

### Blinding and anonymization

Each participant was assigned a random study number. The gas chromatograph operator was blinded to the subject identity and sampling time but, for calibration purposes, knew whether samples were ambient or alveolar. Aggregate data are presented instead of specific age, date or flight duration to prevent attributability.

### Standard of care and ethics

Ethics approval was obtained from the National Aeronautics and Space Administration (NASA) Human Research Multilateral Review Board (#Pro1283) and the Ottawa Health Sciences Research Ethics Board (#2009646-01H). The trial was registered at NASA’s Life Sciences Data Archives (https://lsda.jsc.nasa.gov/Experiment/exper/13399). All participants signed an informed consent form.

### Assumptions and control for confounding

Preflight measures ensured that astronauts had no baseline anemia or hemolysis. Ambient [CO] varies widely. Given the higher and fluctuating ambient [CO] on the ISS, determining hemolysis based only on alveolar [CO] would have grossly overestimated CO elimination in space (Fig. 1c). In this trial, ambient air samples in the immediate environment of the astronaut were collected with each alveolar air sample. While altered pulmonary ventilation in space constitutes a potential unmeasured confounder, previous experiments reported uniform ventilation and similar ventilation/perfusion ratio in space as on Earth^29^. Disrupted circadian cycle in orbit (16 sunrises and sunsets per 24 h) may affect hemolysis^30^. We controlled for this potential confounder by chronostandardizing the sampling according to the astronaut schedule set on Greenwich meridian time. Nonhemoglobin heme proteins (cytochromes and myoglobin) are catalyzed by heme oxygenases to generate 15% of the human CO^6^, but this has never been measured in space. No liver or muscle necrosis was reported in space^31^, and muscle atrophy in space was addressed through exercise countermeasures. Earth-based data on muscle atrophy showed negligible changes in muscle myoglobin content^32,33^. Finally, myoglobin content is approximately eightfold less than hemoglobin content, decreasing the likelihood that nonhemoglobin heme degradation biased the interpretation of CO elimination data^32^. Entering space triggers a 10–12% blood volume contraction due to fluid shifts, which leads to hemoconcentration^1,2,5,34^. We did not apply a 10–12% correction to the initial ISS data, because serum analytes have short half-lives. In addition, Haldane’s equation determining O_2_ and CO binding to hemoglobin is unaffected by hemoconcentration. Importantly, ISS ambient pressure reproduces atmospheric pressure, validating Henry’s law governing CO elimination according to partial pressure gradients.

### Statistical analyses

For this exposure study, summary statistics are limited to point estimates with unadjusted 95% CI widths. No *P* values are provided. We also present mean percent increases from baseline. Because a few samples were damaged, the ratio of estimates at individual time points may differ slightly from the percent increase estimates based on paired observations. Paired observations were necessary to calculate CIs on percent increase.

### Reporting Summary

Further information on research design is available in the Nature Research Reporting Summary linked to this article.

## Online content

Any methods, additional references, Nature Research reporting summaries, source data, extended data, supplementary information, acknowledgements, peer review information; details of author contributions and competing interests; and statements of data and code availability are available at 10.1038/s41591-021-01637-7.

## Supplementary information

Supplementary Tables 1–5 Hemolysis and Erythrocyte markers

Reporting Summary

Supplementary Video Methods to harvest alveolar air.

## Data Availability

Aggregated data to understand and assess the conclusions of this research are available in the figures and supplementary tables. Individual astronaut source data have been deposited in NASA’s Life Sciences Data Archives. Investigators can request access to the astronaut data at https://lsda.jsc.nasa.gov/.
